# Exploring the early origins of the synapse by comparative genomics

**DOI:** 10.1098/rsbl.2008.0594

**Published:** 2008-12-02

**Authors:** Kenneth S. Kosik

**Affiliations:** Neuroscience Research Institute, University of CaliforniaSanta Barbara, CA 93106-5060, USA

**Keywords:** demosponge, porifera, *Amphimedon queenslandica*, post-synaptic scaffold proteins, PDZ domain

## Abstract

One set of evolutionary features that has received less attention than the evolution of genes or species is the evolution of cellular machines, the self-contained structures in cells with dedicated functions. Here I suggest that domain expansion through shuffling, duplication, and changes in protein expression level are critical drivers in the evolution of cellular machines. Once established, evolutionary change in these cellular machines tends to occur by paralogy or expansion and modification of the existing core genes. A comparative genomics approach to one cellular machine—the post-synaptic complex—provided preliminary validation of these views. A comparative genomics approach to the entire cellulome may reveal the diversity of cellular machines and their inter-relationships.

In the hierarchical organization of organisms, the self-assembly of proteins is a fundamental step towards complexity. Protein complexes, often in conjunction with membranous elements or nucleic acids, form a variety of organelles and other subcellular structures that are vital for the lifestyle of the organism. Many of these subcellular structures are described generically with the implicit assumption that they are similar across very broad phylogenetic distances. Indeed, the core components of organelles such as the proteasome, the nuclear pore, the spliceosome, the RNA-induced silencing complex, the adherens junction, and the synapse, to name but a few, are highly conserved. The self-assembly and structural integrity of these subcellular units arises from interaction domains that are among the most highly conserved regions of the protein components. The fit and fitness of interaction domains places a powerful constraint on evolutionary change within these cellular machines.

What belies a relative uniformity among these cellular structures is the pressure of change at levels of the biological hierarchy, which operate outside the confines of the machine. Mutations in the genes, which encode structural proteins, the developmental context from which subcellular structures assemble and organismal change, are among the many factors that affect these machines and may contribute to their adaptive diversification. All the while, function must remain intact. Cellular machines cannot shut down for repairs, upgrades or patches while evolutionary change occurs around them. These considerations lead to the following questions: (i) how diverse are the structural components of cellular machines? and (ii) how does evolutionary change modify cellular machines while maintaining their fundamental structure and function?

In approaching these questions, we focused first on the comparative genomics of those genes that encode the structural components of the post-synaptic junction (Sakarya *et al.* 2007). A related approach was reported by Gabaldon and colleagues with regard to the evolution of NADH : ubiquinone oxidoreductase (complex I) (Gabaldon *et al.* 2005). We catalogued a set of key post-synaptic genes from representative species that branch over long time periods within the animal kingdom. Using the genome of the demosponge, *Amphimedon queenslandica*, the cnidarian *Nematostella vectensis*, *Drosophila melanogaster*, a representative protostome, and *Homo sapiens*, a representative deuterostome, we assembled phylogeny trees for the set of post-synaptic complex genes. The species were selected because they represent major phylogenetic intervals across which the innovations of interest took place.

From these data, it was apparent that the genes which encode the core components of the post-synaptic density are present throughout the entirety of the animal kingdom. Therefore, at first pass, the enormous growth of the brain was not associated with major gene gain or loss events in the post-synaptic density. More recently, we have extended this analysis to the pre-synaptic element, which also retains a set of core genes throughout the entirety of animal evolution (C. Conaco & K. S. Kosik 2008, unpublished observations). The exception is the sponge, *A. queenslandica*, a basal animal, which does not possess synapses or neurons. Interestingly, even in the absence of any recognizable synaptic structure, *A. queenslandica* possesses a nearly complete set of post-synaptic scaffolding genes as well as proneural atonal-related bHLH genes coupled with Notch–Delta signalling (Richards *et al.* 2008). What is lacking for a complete post-synaptic complex are the ionotropic glutamate receptors (iGluR), which belong to the NMDA/AMPA/Kainate receptor families, neuroligin and the Shaker-type voltage gated K^+^ channel. These ‘missing genes’ are present in the cnidarian, *N. vectensis*, as well as all other animals, but absent from the eukaryotic genomes of yeast, *Tetrahymena*, and *Dictyostelium*. From the roll call of *A. queenslandica* synaptic genes, the sponge is missing a set of receptor and channel genes that encode proteins that link the sub-synaptic scaffold to an extracellular input in the synaptic cleft. The missing genes all bind to the synaptic scaffold and form a set of diverse receptors on the post-synaptic membrane which are essential for the complex transduction of the pre-synaptic signal. Whether these genes were gained in Cnidaria or lost in the sponge is unknown.

Those ‘post-synaptic’ genes that are present in *A. queenslandica* are likely to be assembled into a complex structure of unknown function in the sponge and may have been exapted in the evolution of the synapse (Sakarya *et al.* 2007). Although many details need to be filled in, organisms with post-synaptic densities have changed little with respect to the core features of the scaffolding element. The proto-synaptic structure may have been lifted from a metazoan ancestor, and with only minimal tinkering on the core structure added new gene products to create a novel cellular machine. However, positioning the sponge among the earliest branching events of animals remains imprecise. Recently, sequence evidence suggested that ctenophores (comb jellies) may be the sister group to all other sampled metazoans (Dunn 2008). Both cnidarians and ctenophores have synapses and neurons organized as a nerve net. If the sponge branched from an ancestor shared with ctenophores, we might conclude that the modern sponge underwent massive gene loss. Depending on the position of the ctenophores, the evolutionary stem leading to synapses is a common ancestor of all animals or is restricted to descendants from a branch that separated ancestral sponges and all other animals. Thus, modern sponges, which lack synapses and neurons, have either lost their nervous system, or the neural-like structures of ctenophores arose independently of other metazoans. In either case, the sponge resides in a highly informative position with regard to the evolutionary innovations that led to the neuronal synapse.

The events which lead to the putative structure(s) in the sponge with its many post-synaptic scaffolding proteins must have required changes in binding events. Pre-existing scaffold proteins acquired or lost ligands for their binding domains. Examples of genes in *A. queenslandica* with nearly identical domain organizations from sponge to human include a *dlg* family member orthologous to PSD-95, and orthologues of S-SCAM, Homer, Shank and GRIP (Sakarya *et al.* 2007). The presence of novel ligands, neither the introduction of novel domains nor the reorganization of existing domains, characterizes the difference between the sponge and other animals. New binding partners may create competitive inhibition, steric exclusion or cooperativity, which could be parametrized in terms of a Gibbs free energy derived from a systematic characterization of possible complexes. These changes must maintain the stability of the structure and the added functionality of the machine. Examples abound. Two post-synaptic gene homologues in *Amphimedon*, GKAP and Citron lack a PDZ ligand sequence, although other metazoans include ligand sequences in their orthologous genes. These genes either acquired or lost novel binding features. If the sponge complex is ancestral to the synapse, the inclusion of GKAP and Citron in the post-synaptic complex added new binding partners to the pre-existing scaffold. AMPAR binds to GRIP; however, *Amphimedon* lacks AMPAR, but has a GRIP orthologue. This sponge GRIP orthologue includes a PDZ domain that clades with the orthologous GRIP PDZ domain, which binds AMPAR, a clear example of a PDZ domain enjoying a natural niche without one of its ligands. Stargazin, which links AMPAR to PSD-95, is not found in any invertebrate genomes, and therefore represents a more recent innovation that had to compete with the pre-existing binding partners. In fact, the set of post-synaptic genes missing from the sponge all bind to PDZ domains within proteins that have exact orthologues in the sponge. Presumably, these PDZ domains were occupied with pre-existing ligands, and therefore evolutionary selection appears to have operated on the binding kinetics of the scaffold.

Successful interactions added functionality and complexity to the machine. A series of binary events that could result in the assembly of a complex with four pre-existing proteins called A, B, C, D may follow pathways as follows.

(1) A+B→ABandC+D→CD,thenAB+CD→ABCD,

(2) A+B→AB,thenAB+C→ABC,thenABC+D→ABCD.

As subcellular machines increase in complexity, their histories could be represented in a lineage tree that leads to a recognizable annotated complex such as the post-synaptic complex. Presumably, ancestral complexes serve some adaptive function that may or may not be related to the function of the observed complex.

Many of the protein–protein interactions within a cellular machine are kinetically skewed towards the bound state, so that the machine remains assembled. Dissociation of critical protein partners could disable the machine. Stable interactions of this sort are often mediated by compact sequences within proteins called domains. Domain shuffling, which refers to the duplication of a domain or the insertion of a domain from one gene into another, may be an important contributor to phenotypic complexity. If one imagines a lineage tree for a subcellular structure, its evolutionary path would probably depart from the binary events in the histories noted above. Rather, domain shuffling allows the rapid generation of multiple interaction partners. For example, the introduction of three tandem binding domains into a protein, A, will lead to a scaffolding complex in which ligands (B, C, D) for all of these domains are joined in a single step. This third pathway of complex assembly is as follows:

(3) A+B+C+D→ABCD.

If protein A can exist as a dimer, then the number of protein components doubles. Other ligands (X, Y, Z) with affinities, which are competitive with A, B, C, would diversify the composition of the scaffolds. The expression levels of all these ligands as well as the level of scaffold expression will drive mass action effects and greatly affect the distribution of scaffold compositions, each with a distinct array of ligands.

Current genomic data allow one to track synaptic genes not only in basal animals such as sponges that lack synapses, but also prior to the evolution of animals themselves. Choanoflagellates are the closest known relatives of metazoans. In contrast to *A. queenslandica*, the unicellular choanoflagellate *Monosiga brevicollis* genome contains very few orthologues of the post-synaptic gene set (King 2008); however, the domains found within the post-synaptic gene are present in a large variety of other contexts. Indeed, comparative sequence analyses have led to the view that tracing domain phylogeny in proteins may more accurately represent the process of gene/protein evolution than entire proteins (Koonin *et al.* 2000). As concluded in the report on the *M. brevicollis* genome, the physical linkages among protein domains often differ between *M. brevicollis* and metazoans. Abundant domain shuffling at the origin of Metazoa resulted in a set of synaptic genes that have retained their domain organization throughout all metazoan lineages. The domain organization of most post-synaptic genes became fixed in the sponge either before the origination of synapses in organisms derived from a common ancestor of the sponge and other animals, or was retained when synapses were lost in the sponge.

For example, multiple sequence alignments and phylogenetics of PDZ domains can group this domain into distinct clades with a most recent common ancestor that pre-dates metazoans (Sakarya *et al.* 2007, 2008). The unique sequence identities of individual PDZ domains make it possible to distinguish the history of each PDZ domain in its conserved position within the larger protein. The *dlg* gene contains three PDZ domains that can be individually claded and the results of this PDZ domain phylogeny suggest the ancient origin of each in a common ancestor that antedated animals. Thus while PDZ domains can be traced back to prokaryotes, the specialization of PDZ domains within a larger motif organization represents a major Neoproterozoic innovation. The specification of PDZ domains by changes in their sequence and organization within a larger domain architecture very likely happened in a period characterized by a series of cladogenic events closely spaced in time near the origin of Metazoa (Rokas *et al.* 2005).

Over the course of animal evolution, the core set of synaptic genes has neither incurred significant gene loss nor gene gain. What has happened is that the set of core synaptic genes has undergone paralogous expansion. For example, the PSD-95 family is represented by a single gene in *M. brevicollis* and in invertebrates, but in vertebrates expanded to include PSD-95/SAP90, PSD-93/chapsyn-110, SAP-97/hDLG and SAP-102. Diversification has occurred in the region N-terminal to the first PDZ domain, the region between PDZ2 and PDZ3, and the inter-SH3/GK region. Among the functions attributed to these regions of diversification are closely spaced cysteine residues that undergo palmitoylation for differential targeting or differential inclusion of an L27 protein–protein interaction motif for head-to-head multimerization (Hsueh *et al.* 1997; El-Husseini *et al.* 2000; Chetkovich *et al.* 2002; Christopherson *et al.* 2003). Paralogous expansion appears to be an important means by which synapses have evolved within the animal kingdom. Alternatively, paralogy may dominate over even longer time periods, but any residual similarity among ancient, highly divergent proteins has fallen below detection levels. Another important trend observed in animal phylogeny is an increasing number of microRNAs (miRNAs) (Grimson *et al.* 2008). While *M. brevicollis* lacks miRNAs, *A. queenslandica* has eight miRNAs, *N. vectensis* has 40 miRNAs, the planarian, *Schmidtea mediterranea* has 61 miRNAs, *Caenorhabditis elegans* has 154 miRNAs, *D. melanogaster* has 147 miRNAs, *Mus musculus* has 491 miRNAs and humans have 677 miRNAs. The ability to regulate sets of genes post-transcriptionally may have also contributed to synaptic evolution.

The deep organizational structure of the synapse may be better understood through its evolutionary origins. As noted, sponges appear to have a protein complex that uses orthologues of true metazoan synaptic genes and could have a quasi-neural role in mediating long distance communication and coordinated behaviours. For example, the freshwater sponge *Ephydatia muelleri* (Demospongiae, Haplosclerida, Spongillidae) is capable of peristaltic-like contractions to expel waste material from its aquiferous system (Elliott and Leys 2007). More importantly, in terms of synapse evolution, a sponge ‘proto-synapse’ would suggest that latent within the synapse lies an ancient relic capable of functioning on its own. Techniques, such as knockouts or RNAi, that alter single genes are limited in yielding the underlying logic of a biological system, whereas comparative genomic techniques could reveal these unsuspected functional units.

One can generalize this comparative genomic approach to all subcellular structures as the study of the cellulome. Systematically cataloguing the constituent sets of proteins across many species for each subcellular structure opens a wealth of experimentally tractable questions. We can ask how much species variation exists among a subcellular structure in its protein components and its use of paralogues to diversify structure and extend function. We can ask how new proteins arising from gene innovation get integrated within pre-existing subcellular machines. From these data, we can ask how biological machine evolution tracks with the species tree. We can ask whether smaller units within these machines can be fitted into other machines and whether machine parts jump into new machines or reorganize to invent new machines.

Cataloguing this paralogy is the subject of the cellulome.
